# Bile acid-FXRα pathways regulate male sexual maturation in mice

**DOI:** 10.18632/oncotarget.7153

**Published:** 2016-02-03

**Authors:** Marine Baptissart, Emmanuelle Martinot, Aurélie Vega, Lauriane Sédes, Betty Rouaisnel, Angélique de Haze, Silvère Baron, Kristina Schoonjans, Françoise Caira, David H. Volle

**Affiliations:** ^1^ INSERM U 1103, Laboratoire GReD, Campus Universitaire des Cézeaux, TSA 60026, CS 60026, 63178 Aubière Cedex, France; ^2^ Université Clermont Auvergne, Université Blaise Pascal, GReD, F-63178 Aubière, France; ^3^ CNRS, UMR 6293, GReD, F-63178 Aubière, France; ^4^ Centre de Recherche en Nutrition Humaine d'Auvergne, F-63000 Clermont-Ferrand, France; ^5^ Institute of Bioengineering, Ecole Polytechnique Fédérale de Lausanne, CH-1015 Lausanne, Switzerland

**Keywords:** testicular steroidogenesis, nuclear receptors, hypothalamo-pituitary axis, bile acid, germ cell apoptosis

## Abstract

The bile acid receptor Farnesol-X-Receptor alpha (FRXα) is a member of the nuclear receptor superfamily. FRXα is expressed in the interstitial compartment of the adult testes, which contain the Leydig cells. In adult, short term treatment (12 hours) with FRXα agonist inhibits the expression of steroidogenic genes *via* the induction of the Small heterodimer partner (SHP). However the consequences of FRXα activation on testicular pathophysiology have never been evaluated. We demonstrate here that mice fed a diet supplemented with bile acid during pubertal age show increased incidence of infertility. This is associated with altered differentiation and increase apoptosis of germ cells due to lower testosterone levels. At the molecular level, next to the repression of basal steroidogenesis via the induction expression of *Shp* and *Dax-1*, two repressors of steroidogenesis, the main action of the BA-FRXα signaling is through lowering the Leydig cell sensitivity to the hypothalamo-pituitary axis, the main regulator of testicular endocrine function. In conclusion, BA-FRXα signaling is a critical actor during sexual maturation.

## INTRODUCTION

The nuclear receptor Small Heterodimer Partner (SHP) have been demonstrated to control sexual maturation in male mice [1]. SHP is a known target gene of the nuclear bile acid receptor Farnesol-X-Receptor-α (FRXα). However, the potential roles of BA on male sexual maturation have never been studied so far. Interestingly, experimental models of liver injury show altered puberty with primary hypogonadism [2]. It is known that such conditions of liver disorders lead to increased bile acid levels. We hypothesized that BAs could alter male sexual maturation during puberty *via* FRXα.

Puberty is a key event for the establishment of male reproductive functions. Puberty depends on the increase of testosterone levels which is under the control of the hypothalamo-pituitary axis activity. This leads to the maturation of secondary sexual characteristics, the establishment and the maintenance of spermatogenesis and then fertility [3].

In the present study, we first analyzed the impact of pubertal BA-exposure on testicular physiology. In order to decipher the involved molecular mechanisms, we used a classical approach with diet supplemented with BA (cholic acid 0.5%) [4]. We demonstrate that pubertal mice fed a diet supplemented with cholic acid (CA) have altered fertility associated with default in germ cell differentiation correlated with an increased rate of spermatocytes apoptosis. We have validated that these effects are not mediated by TGR5. This is due to altered testosterone synthesis. Surprisingly these impacts of BA were not fully mediated by SHP. Interestingly, we pinpoint that the gene encoding the nuclear receptor *Dax-1* (dosage-sensitive sex reversal, adrenal hypoplasia critical region, on chromosome X, gene-1), a repressor of steroidogenesis and a related to SHP, is a target of FRXα. Moreover, we defined a major impact of BA-exposure explaining the impact on testicular endocrine function. Indeed, in vivo and in vitro approaches demonstrated that FRXα activation decreases Leydig cells sensitivity to the hypothalamo-pituitary axis signaling. Using pharmacological experiments we have established that the effect of BAs is mainly due to the transcriptional repression of the gene encoding the luteinizing hormone receptor (Lhcgr). BA levels are increased during liver diseases, thus these results, in combination with previous study in adult, highlight the complexity of the interaction between the liver and testicular functions throughout lifetime.

## RESULTS

### Dietary BA supplementation alters male fertility

**Figure 1:**
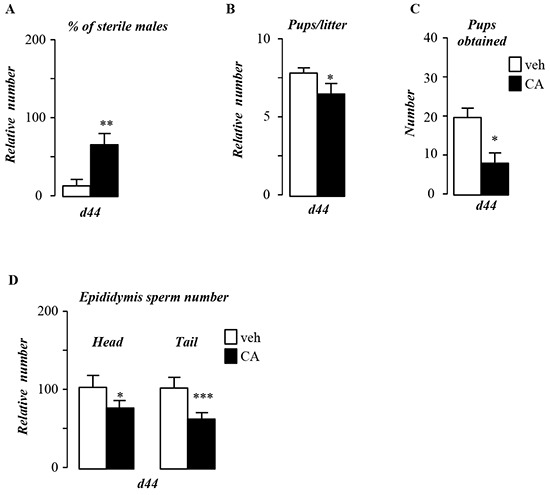
Pubertal exposure to BA alters male fertility **A.** Percentage of infertile males. **B.** Number of pups per litter. **C.** Total number of pups obtained per group. **D.** Sperm count in the epididymis head and tail of control or CA fed groups for 44 days. In all of the panels data are expressed as the means ± SEM. Statistical analysis:*, p<0.05; **, p<0.01, ***, p<0.005 *vs.* control diet group.

To identify links between pubertal BA-exposure and male fertility, mice were fed a normal control diet supplemented with 0.5% of cholic acid (CA). CA-diet led to altered fertility with 60% of the exposed males unable to give progeny (Figure 1A). In males giving progenies, CA-diet also decreased the number of pups per litter (Figure 1B). The combination of the increase of sterile males and decrease of pups per litter resulted in a 60% decrease of the number of pups generated by CA-exposed males compare to control-diet group (Figure 1C). This decreased fertility was associated with a lower production of spermatozoa as revealed by the counting of sperm number in the epididymis head and tail (Figure 1D).

### BA-diet alters postnatal growth

**Figure 2:**
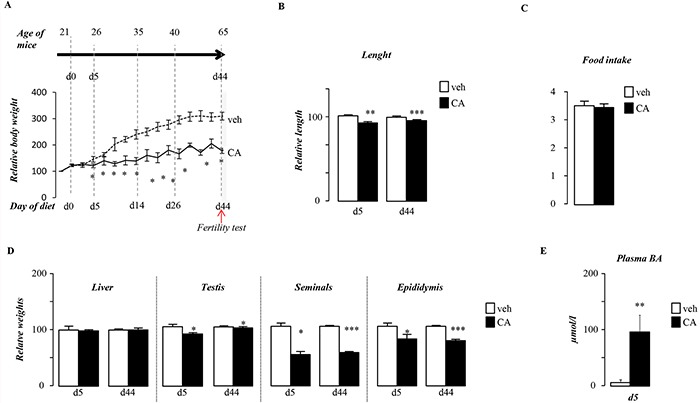
Pubertal exposure to BA alters pubertal growth **A.** Overall body weight gain of males exposed to control or CA diets. **B.** Body length of males after 5 or 44 days of either control or CA diet. **C.** Overall food intake of male fed control or CA diet. **D.** Liver, testis, epididymis and seminal relative gross weights in C57Bl/6J mice fed 5 or 44 days of exposure. **E.** Plasma bile acid levels in males exposed to control or CA diets for 5days. In all of the panels data are expressed as the means ± SEM. Statistical analysis: *, p<0.05; **, p<0.01, ***, p<0.005 *vs.*respective control group.

The impact of BA-diet on fertility is associated with altered postnatal growth as mice exposed to CA-diet present lower weight gain starting 5 days after the beginning of the treatment (Figure 2A). CA-exposed males showed reduced body length (Figure 2B). However, the overall food intake was not altered (Figure 2C). The altered postnatal growth during this pubertal period affects male genital tract with lower weight gain in testis, epididymis and seminals (Figure 2D), whereas the liver weight was not affected (Figure 2D). In addition, it must be noticed that the liver weight relative to body weight was increased suggesting liver injury (Supplementary Figure S1A); which is consistent with alteration of genes such as Cyp3a25 and Sult2a1 (Supplementary Figure S1B) and the increase of plasma BA levels after 5 days of CA-diet exposure (Figure 2E). In Figure 2D, weights are represented with chow-diet group as reference (100%) at particular age. It has to be noticed that organ gross weights increased during postnatal development (between 5 days post-treatment (dpt) and 44dpt), the increase is less pronounced in CA-diet treated mice (Supplementary Figure S1C) which sustained the impact of CA-diet on global postnatal growth. In addition, weights relative to body weight of epididymis and seminals was also affected (Supplementary Figure S2D).

### Pubertal BA-exposure alters germ cell survival

**Figure 3:**
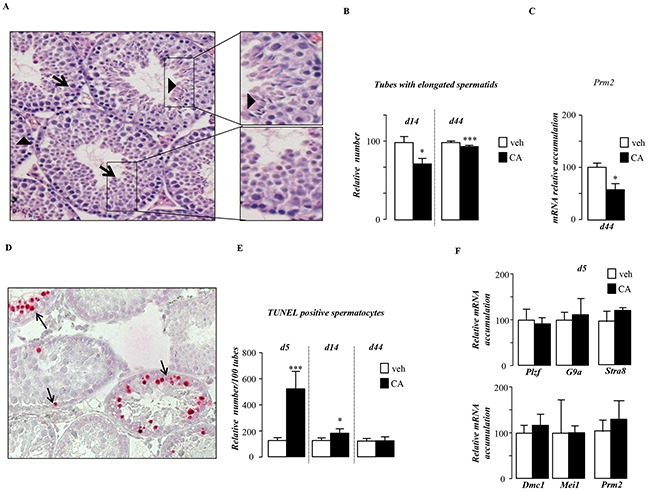
Pubertal exposure to BA alters germ cell survival **A.** Representative micrographs of hematoxylin/eosin-stained testes of mice fed CA-diet for 14 days. The arrow-head indicates tubules with elongated spermatids; arrows indicate tubes without elongated spermatids. The original magnification was x200. **B.** Quantification of the number of tubules with elongated spermatids per 100 seminiferous tubules after 14 and 44 days of control or CA-diet (n=10-20 per group). **C.** Testicular mRNA expression of *Prm2* normalized to *β-actin* levels in whole testis of C57Bl/6J mice fed control or CA diets for 44 days (n=10 to 15 per group). **D.** Apoptosis in mice exposed to control or CA diets (n=10-20 per group) analyzed by TUNEL staining. Representative micrographs of testis exposed to control or CA diets for 5 days. The arrows indicate apoptotic spermatocytes. The original magnification was x200. **E.** Quantification of TUNEL analyses after 5, 14 or 44 days of diet exposure. The number of TUNEL-positive is indicated as the number of positive cells per 100 seminiferous tubules (n=10-20). **F.** Testicular mRNA expression of *Plzf, G9a, Stra8, Dmc1, Mei1* and *Prm2* normalized to *β-actin* levels in whole testis of C57Bl/6J mice fed control or CA diets for 5 days (n=10 to 15 per group). Control diet treated mice were arbitrarily fixed at 100%. In all of the panels data are expressed as the means ± SEM. Statistical analysis: *, p<0.05; **, p<0.01, ***, p<0.005 *vs.*respective control group.

Histological analyses of testis showed that BA-exposed mice showed altered germ cell differentiation as visualized with a decrease in the number of seminiferous tubules with elongated spermatid cells (Figure 3A & 3B). Analysis of the expression of pre-meiotic (*Plzf*, *G9a, Stra8*,*),* meiotic (*Dmc1*, *Mei1)* and post-meiotic (Prm2) genes showed peculiar kinetic of events. Consistent with the observed decrease of post-meiotic cells at 44dpt (day post beginning of the treatment) the expression of *Prm2* was lower in CA-treated group compare to control group (Figure 3C). Interestingly at this age the pre-meiotic and meiotic genes were not affected at that age (Supplementary Figure S2A). This delay in germ cell differentiation was correlated with an early apoptotic wave of germ cells after 5-days of CA-diet (Figure 3D & 3E). This apoptotic wave was transient as at 44-days after the beginning of the treatment no more difference was observed between groups (Figure 3E). In contrast, 5 days after the beginning of the treatment, neither the mRNA accumulation of pre-meiotic and meiotic genes such as *Plzf*, *G9a*, *Stra8*, *Dmc1* and *Mei1* nor the post meiotic gene, Prm2 were significantly affected by the CA-diet (Figure 3F). Thus the altered meiosis process was not due to the altered expression of key meiotic genes such as *Stra-8* and *Dmc1.*

### BA-diet induces germ cell death via alteration of testosterone metabolism

**Figure 4:**
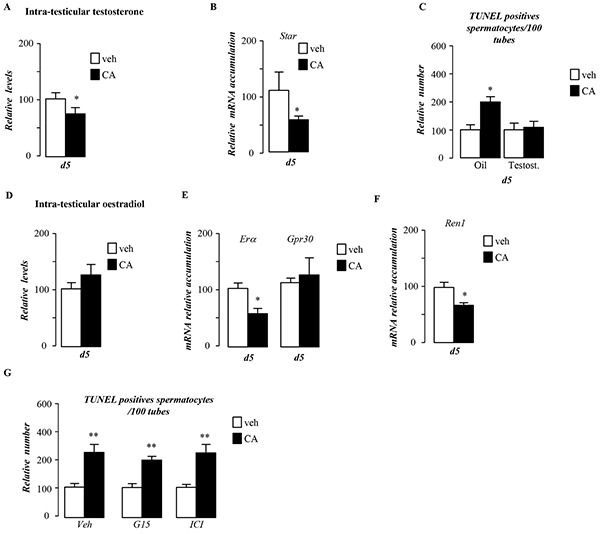
Pubertal BA exposure regulates testicular androgen metabolism **A.** Relative intra-testicular testosterone levels in C57Bl/6J mice fed control or CA diet for 5 days (n=6-20 per group). **B.** Testicular mRNA expression of *Star* normalized to *β-actin* levels in whole testis of C57Bl/6J mice fed control or CA diets for 5 days (n=10 to 15 per group). **C.** Quantification of TUNEL analyses after testosterone or vehicle treatment in males fed control or CA diets for 5 days. The number of TUNEL-positive spermatocytes is indicated as the number of positive cells per 100 seminiferous tubules (n=10-20). **D.** Relative intra-testicular estrogen levels in C57Bl/6J mice fed control or CA diet for 5 days (n=6-20 per group). **E.** Testicular mRNA expression of *Erα* and *Gpr30* normalized to *β-actin* levels in whole testis of C57Bl/6J mice fed control or CA diets for 5 days (n=10 to 15 per group). **F.** Testicular mRNA expression of *Renin-1* normalized to *β-actin* levels in whole testis of C57Bl/6J mice fed control or CA diets for 5 days (n=10 to 15 per group). **G.** Quantification of the number of TUNEL positive cells per 100 seminiferous tubules after 5 days of control or CA-diet co-treated with either vehicle, G15 or ICI (n=5-10 per group). In all panels control diet or vehicle treated group were arbitrarily fixed at 100% and data are expressed as the means ± SEM. Statistical analysis: *, p<0.05; **, p<0.01, ***, p<0.005 *vs.* respective control group.

Germ cell death has previously been associated with androgen withdrawal [5]. Interestingly, CA-exposed males, for 5 days, showed a decrease of intra-testicular levels of testosterone (Figure 4A). This was associated with a decrease of the *Steroidogenic acute regulatory protein* (*Star)* mRNA accumulation in testis of CA-exposed mice compare to control group (Figure 4B). No statistically significant effect was found on testosterone levels after 44 days of CA-diet (Supplementary Figure S2B), whereas star mRNA accumulation was still decrease in CA-exposed males (Supplementary Figure S2C). The involvement of testosterone decrease in germ cell death was sustained by the fact that supplementation with testosterone counteracted the effect of CA-diet on germ cell apoptosis (Figure 4C). In order to decipher if testosterone impacted *per se* germ cell physiology or if it could act *via* its aromatization into estradiol, we studied the estrogenic signaling pathways, which are known regulators of steroidogenesis in Leydig cells [6], [7]. Intra-testicular levels of estradiol were not affected by CA-diet after 5-days of exposure (Figure 4D), a time when germ cell apoptosis was seen. We next analyzed the expression of the two estrogen receptors described in Leydig cells, the G protein coupled receptor *Gpr30* [7] and the nuclear receptor estrogen receptor alpha (*Erα*) [8]. If the expression of *Gpr30* was not altered, the CA-exposed males showed a lower level of *Erα* compared to control group (Figure 4E). This impact on *Erα* expression was supported by the altered expression of testicular ERα target genes such as Renin-1 (Figure 4F). These results suggest that estrogenic pathway must be altered in the context of CA-diet. In order to discriminate its involvement in CA-induced germ cell apoptosis, we used specific antagonists of GPR30 or ERα, respectively G15 [9] and ICI 182, 780 [10]. None of these antagonists was able to counteract the effect of CA on germ cell death (Figure 4G).

### BA-diet alters germ cell survival in TGR5 independent pathways

**Figure 5:**
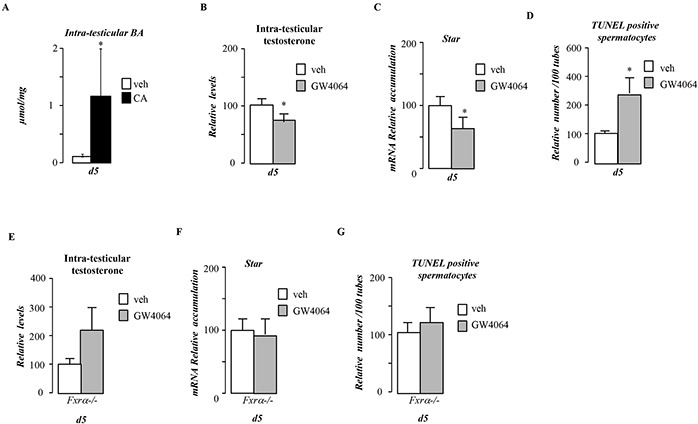
Pubertal BA exposure impacts testis physiology via FXRα **A.** Relative intra-testicular bile acid levels in *C57Bl6.*mice fed control or CA diet for 5 days (n=5-6 per group). **B.** Relative intra-testicular testosterone levels in *C57Bl6.*mice treated with vehicle or GW4064 for 5 days (n=5-6 per group). **C.** Testicular mRNA expression of *Star* normalized to *β-actin* levels in whole testis of C57Bl/6J mice treated with vehicle or GW4064 for 5 days (n=10 to 15 per group). **D.** Quantification of TUNEL analyses in males treated with vehicle or GW4064 for 5 days. The number of TUNEL-positive spermatocytes is indicated as the number of positive cells per 100 seminiferous tubules (n=10-20). **E.** Relative intra-testicular testosterone levels in *Fxrα−/− male* mice treated with vehicle or GW4064 for 5 days (n=5-6 per group). **F.** Testicular mRNA expression of *Star* normalized to *β-actin* levels in whole testis of *Fxrα−/− male* mice mice treated with vehicle or GW4064 for 5 days (n=10 to 15 per group). **G.** Quantification of TUNEL analyses in *Fxrα−/− male* mice treated with vehicle or GW4064 for 5 days. The number of TUNEL-positive spermatocytes is indicated as the number of positive cells per 100 seminiferous tubules (n=10-20). In all panels control diet or vehicle treated group were arbitrarily fixed at 100% and data are expressed as the means ± SEM. Statistical analysis: *, p<0.05; **, p<0.01, ***, p<0.005 *vs.*respective control group.

Consistent with what was previously demonstrated, exposure to BA-diet led to increase of intra-testicular BA levels (Figure 5A). In contrast to the adult BA exposure [11], our data clearly demonstrate that TGR5 was not involved in the pubertal phenotype induced by BA-exposure as *Tgr5^−/−^* mice showed altered fertility associated with higher apoptotic germ cell (Supplementary Figure S3A, S3B, S3C & S3D) in response to CA-diet. The lack of role of TGR5 is sustained by the fact that the mRNA accumulation of Connexin-43, a testicular TGR5 target gene [11], was not altered in the present model (Supplementary Figure S3E).

### BA-diet alters pubertal testicular physiology through FRXα

Due to the hyper-sensibility of Fxrα−/− mice to CA-diet, these mice could not be exposed to CA-diet as high mortality levels was observed even after 5-days of exposure. Thus to decipher the potential involvement of FXRα in the impact of BA-diet, males were thus exposed to FXRα synthetic agonist, GW4064. 5-days of exposure with GW4064 days repressed testosterone synthesis (Figure 5B) associated with lower *Star* mRNA accumulation (Figure 5C). This in turn led to an increase of germ cell apoptosis (Figure 5D). This supports the idea that FXRα was involved in the observed phenotype. Consistently, no decrease of testosterone levels was observed in mice invalidated for the gene encoding FXRα (Fxrα−/−) in response to GW4064 (Figure 5E). In that line no effect of GW4064 was observed on *Star* mRNA accumulation in Fxrα−/− males (Figure 5F). Moreover, no impact of GW4064 on the number of apoptotic cells was observed in Fxrα−/− mice (Figure 5G).

### FXRα-BA-diet alters germ cell survival in SHP independent pathways

**Figure 6:**
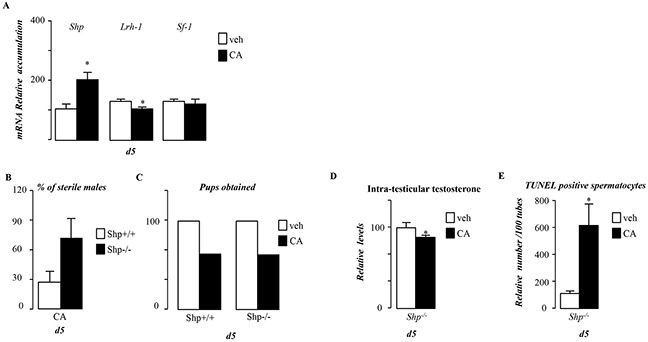
BA act in though FXRα in a SHP independent manner in pubertal males **A.** Testicular mRNA expression of *Shp, Lrh-1*and *Sf-1* normalized to *β-actin* levels in whole testis of C57Bl/6J mice fed control or CA diets for 5 days (n=5 to 10 per group). **B.** Percentage of infertile males in Shp+/+ and Shp−/− exposed to CA diet for 44 days. **C.** Total number of pups obtained per group in Shp+/+ and Shp−/− exposed to control or CA diet for 44 days. **D.** Relative intra-testicular testosterone levels in Shp−/− mice fed control or CA diet for 5 days (n=6-10 per group) **E.** Quantification of TUNEL analyses in Shp−/− males fed control or CA diets for 5 days. The number of TUNEL-positive spermatocytes is indicated as the number of positive cells per 100 seminiferous tubules (n=6-10). In all of the panels data are expressed as the means ± SEM. Statistical analysis:*, p<0.05; **, p<0.01, ***, p<0.005 *vs.*respective control group.

We next wanted to analyze the involvement of the small heterodimer partner receptor (SHP), a known target gene of FXRα, which has been demonstrated to repress steroidogenesis [1]. In the present study, *Shp* expression was increased by CA-diet during pubertal period (Figure 6A). Consistent with previous work the increase of *Shp* was associated with a lower accumulation of *Lrh-1* mRNA, a known inducer of steroidogenesis (Figure 6A). In contrast, the expression of *Sf1* was not affected (Figure 6A). We studied the role of SHP in the testicular phenotype during juvenile cholestasis using knock-out mice. Surprisingly, *Shp*^−/−^ males exposed to CA-diet showed altered fertility (Figure 6B & 6C). In addition, as in wild-type, CA-diet led to a lower testosterone level (Figure 6D) associated with an increased germ cell apoptosis (Figure 6E) in *Shp*^−/−^ males. This suggests that during puberty, BAs can repress testicular steroidogenesis in a SHP-independent manner.

### Dax-1 is a direct target gene of FXRα/RXR heterodimer

**Figure 7:**
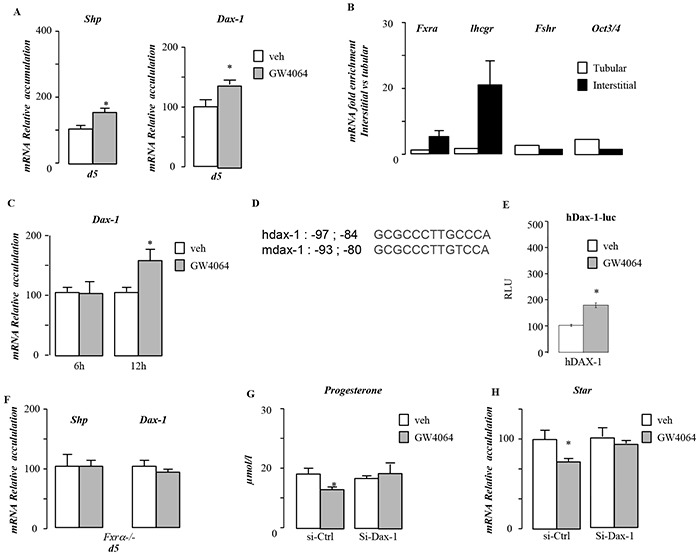
Dax-1 is a target gene of FXRα **A.** Testicular mRNA expression of *Shp* and *Dax-1* normalized to *b-actin* levels in whole testis of C57Bl/6J mice treated with vehicle or GW4064 for 5 days (n=5 to 10 per group). **B.** mRNA expression of *Fxrα*, *Lhcgr*, *Fshr* and *Oct3/4* normalized to *β-actin* levels in interstitial and tubular compartment of 15-days-old testis of C57Bl/6J. **C.** mRNA expression of *Dax-1* normalized to *β-actin* levels in MA-10 cells treated for 6hours or 12 hours with vehicle or GW4064 (n=10 per group). **D.** Sequences of FXRE putative biding site (IR1) in human and mouse DAX-1 promoters. **E.** CV1cells were transfected with pCMX-mFXRα, pCMX-mRXRα, or both receptor plasmids in the presence of the 1.2kbDAX-1 promoter (hDAX1) linked to luciferase. Cells were treated with GW4064 (1 μM). **F.** Testicular mRNA expression of *Shp* and *Dax-1* normalized to *β-actin* levels in whole testis of *Fxrα*−/− mice treated with vehicle or GW4064 for 5 days (n=5 to 10 per group). **G.** Relative progesterone levels in medium of MA10 cells transfected with siRNA-control or siRNA directed against Dax-1 and treated for 12 hours with vehicle or GW4064 (n=6-15 per group). **H.** mRNA expression of *Star* normalized to *β-actin* levels in MA10 cells transfected with siRNA-control or siRNA directed against Dax-1 and treated for 12 hours with vehicle or GW4064 (n=10 to 15 per group). In all of the panels data are expressed as the means ± SEM. Statistical analysis:*, p<0.05; **, p<0.01, ***, p<0.005 *vs.*respective control group.

As *Shp* deficiency was not sufficient to reverse the impact of BA exposure on testosterone synthesis, we wondered whether other repressor of steroidogenesis could be involved. We focused on DAX-1 which is closely related to SHP. In order to validate if *Dax-1* is a *bona fide* target gene of FXRα, we used GW4064. Treatment with GW4064 resulted in an increased of testicular mRNA accumulation of both *Shp* and *Dax-1* (Figure 7A). As Dax-1 is expressed in several cell types of the testis [12, 13], we wanted to ensure that the effect of GW4064 was on Leydig cells. *In vivo* we demonstrate that FXRα is mainly expressed in interstitial compartment of the testis as ensure by the analysis of the expression of specific markers such as *Lhcgr* (Leydig) and *Fshr* (Sertoli) or *Oct3/4* (germ cells) (Figure 7B). As for *Lhcgr*, the expression of *Fxrα* was enriched in interstitial samples. In contrast, *Fshr* and *Oct3/4* only show slight increase in tubular and not in Intestitial samples. The slight increase of *Fshr* and *Oct3/4* might be explained by the fact that Sertoli and Oct3/4 positive spermatogonia represent a small percentage of cells within the seminiferous tubules as these samples contained peritubular cells as well as differentiating spermatogonia, primary and secondary spermatocytes as well as post-meiotic germ cells. MA10 Leydig cells treated with GW4064 showed similar increased of *Dax-1* mRNA accumulation after 12 hours of treatment (Figure 7C). *In silico* analysis of 5′-sequences of the *Dax-1* mouse and human genes revealed a putative FXRE sequence (IR1) (Figure 7D). Ability of FXRα to transactivate the promoter of *hDAX-1* was assessed. Ectopic expression of RXR/FXRα by transient transfection elicited 1.2kb-h*DAX-1* promoter activity in a ligand-dependent manner as shown by the use of GW4064 (Figure 7E). Direct interaction of the RXR/FXRα heterodimer with the FXRE was confirmed by electromobility shift assays. A significant band shift was observed when both FXRα and RXR were added, which was specifically competed away by a 200-fold molar excess of the unlabeled consensus IR1 sequence of FGF19gene ; whereas nonspecific sequence (LXRE [14]) did not compete the binding of the heterodimer. (Supplementary Figure S4A).

Using Fxrα−/− males exposed 5 days to GW4064, *Shp* and *Dax-1* were confirmed as FXRα target within the testis (Figure 7F).

In order to define if DAX-1 is involved in the effect of FXRα on testicular steroidogenesis, we analyzed the impact of GW4064 in MA-10 cells transfected with a siRNA directed against Dax-1. Data show that DAX-1 is involved in the impact of GW4064 on steroidogenesis as supported by the lack of impact of GW4064 on steroid synthesis and *Star* mRNA accumulation in cell treated with a specific siRNA directed against *Dax-1*(Figure 7G & 7H). Combined these data suggest that DAX-1 is involved for the effect of the FXRα-GW4064 impact on basal testicular steroidogenesis.

### BA exposure reduces Leydig cell sensitivity to LH/CG

**Figure 8:**
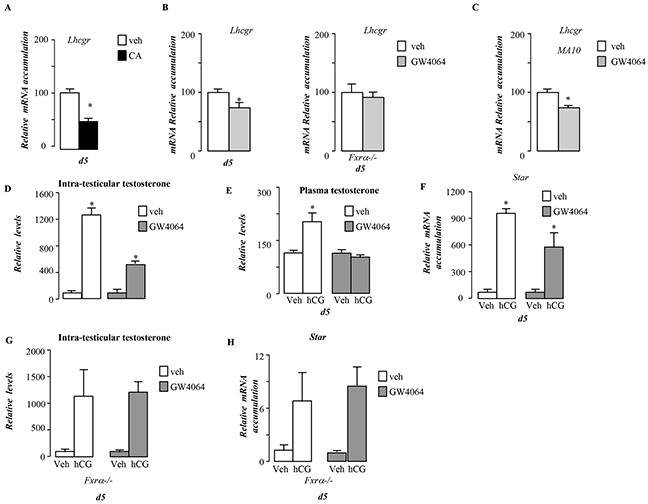
BA signaling pathway alters LH/CG sensitivity in pubertal males **A.** Testicular mRNA expression of *Lhcgr* normalized to *β-actin* levels in whole testis of C57Bl/6J mice fed control or CA diets for 5 days (n=5 to 10 per group). **B.** Testicular mRNA expression of *Lhcgr* normalized to *β-actin* levels in whole testis of C57Bl/6J or *Fxrα*−/− mice exposed to vehicle or GW4064 for 5 days (n=5 to 10 per group). **C.** mRNA expression of *Lhcgr* normalized to *β-actin* levels in MA-10 cells treated for 6 hours with vehicle or GW4064 (n=10 per group). **D.** Relative intra-testicular testosterone levels in C57Bl/6J mice treated 5 days with vehicle orGW4064 and then 12 hours with vehicle or hCG (n=5-15 per group). **E.** Relative plasma testosterone levels in C57Bl/6J mice treated 5 days with vehicle or GW4064 and then 12 hours with veh or hCG (n=5-15 per group). **F.** Testicular mRNA expression of *Star* normalized to *β-actin* levels in whole testis of C57Bl/6J mice treated 5 days with vehicle or GW4064 and then 12hours with veh or hCG. **G.** Relative intra-testicular testosterone levels in *Fxrα*−/− mice treated 5 days with vehicle, GW4064 and then 12 hours with vehicle or hCG (n=5-15 per group). **H.** Testicular mRNA expression of *Star* normalized to *β-actin* levels in whole testis of *Fxrα*−/− mice treated 5 days with vehicle or GW4064 and then 12hours with veh or hCG. In all of the panels data are expressed as the means ± SEM. Statistical analysis:*, p<0.05; **, p<0.01, ***, p<0.005 *vs.*respective control group.

In addition, we demonstrate that *in vivo Lhcgr* mRNA accumulation was altered by FXRα-signaling pathways using either CA-diet or GW4064 exposures (Figure 8A). This effect was mediated by FXRα as no effect of GW4064 on Lhcgr was observed in Fxrα−/− males (Figure 8B). This effect was also observed in MA10 cells (Figure 8C). These data suggest that exposure to FXRα agonist could alter the sensitivity of Leydig cells to luteinizing hormone/chorionic gonadotropin (LH/CG). We tested this hypothesis *in vivo* and *in vitro*. For that purpose, male mice were exposed to GW4064 for 5 days and were injected with 5IU of hCG for 12 hours. In vehicle treated mice, hCG induced a 10-fold increase of testosterone levels within testis (Figure 8D) associated with a 2-fold increase at plasma levels (Figure 8E). In contrast, the GW4064 pre-exposed males showed only a 5 fold increase of testicular levels of testosterone and no elevation in the plasma (Figure 8D & 8E). This was sustained by the lower effect of hCG on *Star* mRNA accumulation in GW4064 treated group compare to vehicle one (Figure 8F).

Interestingly, the lower sensitivity to LH/hCG induced by GW4064 was not observed in Fxrα−/− males as revealed by the measurements of testosterone levels (Figure 8G), *Star* mRNA accumulation (Figure 8H).

**Figure 9:**
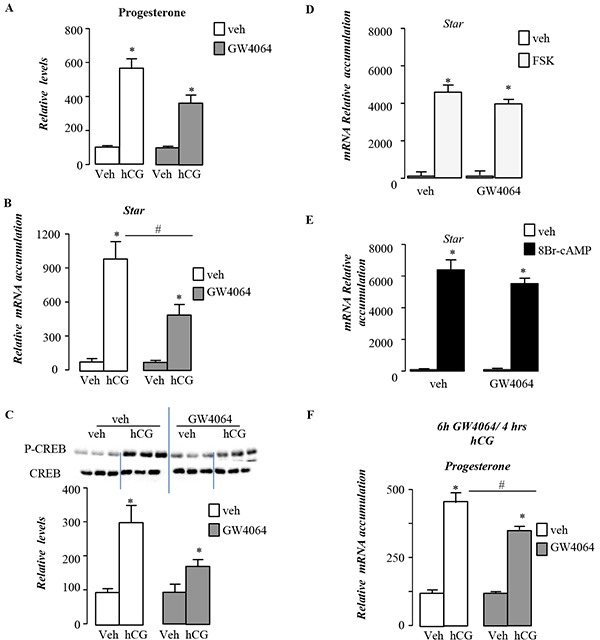
BA signaling pathway alters LH/CG sensitivity at the Leydig cell level in pubertal males **A.** Relative progesterone levels in medium of MA10 cells pre-treated 12h with vehicle or GW4064 and then 4 hours with veh or hCG (n=5-15 per group). **B.** mRNA expression of *Star* normalized to *β-actin* levels in MA10 cells pre-treated 12h with vehicle or GW4064 and then 4 hours with veh or hCG (n=5-15 per group). **C.** Representative western bots of P-CREB and CREB, and quantification of the P-CREB/CREB ratio in MA10 cells MA10 cells pre-treated 12h with vehicle or GW4064 and then 30min with veh or hCG (n=5-15 per group). **D.** mRNA expression of *Star* normalized to *β-actin* levels in MA10 cells pre-treated 12h with vehicle or GW4064 and then 4 hours with veh or Fsk (n=5-15 per group). **E.** mRNA expression of *Star* normalized to *β-actin* levels in MA10 cells pre-treated 12h with vehicle or GW4064 and then 4 hours with veh or 8Bromo-AMPc (n=5-15 per group). **F.** Relative progesterone levels in medium of MA10 cells pre-treated 6h with vehicle or GW4064 and then 4 hours with veh or hCG (n=5-10 per group). In all panels data are expressed as the means ± SEM. Statistical analysis:*, p<0.05; **, p<0.01, ***, p<0.005 *vs.*respective control group.

Consistent with *in vivo* experiments, pre-treatment of MA10 cells with GW4064 for 12 hours decreased the response to hCG with lower sensitivity regarding steroid production in GW4064 condition compare to vehicle treated cells (Figure 9A). This was supported by the lower fold-induction of steroidogenic genes such as *Star* in GW4064 condition compare to vehicle treated cells (Figure 9B). The altered sensitivity of MA10 cells to LH/CG following GW4064 treatment, was supported at the level of the intracellular signaling as measured by the lower increased of CREB phosphorylation in GW4064 treated compare to vehicle group (Figure 9C).

The involvement of the down-regulation of the *Lhcgr* expression in these effects was sustained by the fact that GW4064 was not able to counteract nor the effect of Forskolin (Figure 9D), an adenylate cyclase activator, neither the impact of cAMP analog 8-BromocAMP (Figure 9D). Interestingly, the expression of *Dax-1* was demonstrated to be inhibited by LH/hCG pathway. If GW4064 counteracted the effect of LH/hCG on *Dax-1* mRNA accumulation, it had no effect on the impact of FSK and 8Bromo-cAMP on Dax-1 (Supplementary Figure S4D).

In order to analyze the potential individual or combined roles of DAX-1 and SHP in this lower sensitivity to LH/hCG signaling, we performed experiments using MA-10 cell lines. The decreased sensitivity to LH/hCG was observed after 6h or 12h after GW4064 exposure (Figure 9F). However, the increase of *Dax-1* mRNA accumulations in response to GW4064 treatment was only observed at 12h (Figure 7C) These results support the idea that neither SHP nor DAX-1 were main mediators of the effects of the FXRα-GW4064 effects on LH/hCG stimulated steroidogenesis within Leydig cells. We next tested the impact of GW4064 on known repressor of Lhcgr gene. We tested the expression of Ear2 and Couptf. In vivo data did not allowed determining the molecular mechanisms how FXRα could inhibit Lhcgr expression as Ear2 was not detected on prepubertal testis and Couptf expression was not affected by GW4064 treatment (data not shown).

Finally, we wondered whether this effect of FXRα signaling pathway on LH/hCG stimulated steroidogenesis was specific to pubertal age. In order to answer such question, 12day-old mice were exposed for 5 days to GW4064. At this age, no impact on testosterone levels (Supplementary Figure S5A) or apoptotic germ cell rate was observed in response to GW4064 compare to vehicle-treated group (Supplementary Figure S5B). In addition, the analyses showed that at this age GW4064 altered the sensitivity to LH/hCG. Indeed, chow-treated group showed a 8.6 fold increase of testosterone levels in response to LH/hCG whereas GW4064-treated mice presented only a 2 fold increase (Supplementary Figure S5C). In contrast, at 44-dpt, if the impact of CA-diet on testosterone levels still present, the sensitivity to LH/hCG was no more present on CA-diet exposed mice (Supplementary Figure S5D).

## DISCUSSION

**Figure 10:**
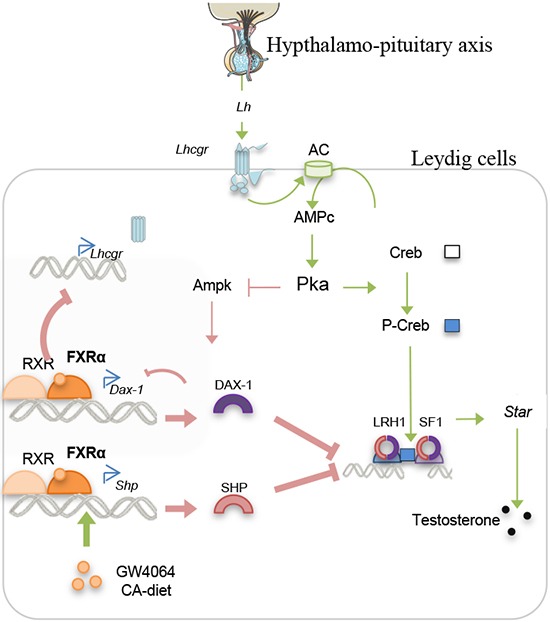
Proposed model for the action of BA-FXRα pathways on Leydig cells during puberty

In the present work, we point out that BAs alter testicular physiology during sexual maturation. We demonstrate that BA exposure could act on testis endocrine function. We provide evidence for the critical roles played by the FXRα-SHP/DAX1 signaling pathways in the regulation of basal steroidogenesis (Figure 10). Moreover, we showed that BA signaling alter the response to the hypothalamo-pituitary axis, the main regulator of endocrine function (Figure 10). This effect seems to be due to the control of the LH receptor expression by FXRα.

One could have thought that systemic effect could be involved on the described phenotype following GW4064 or CA-diet. In similar way of the intestine/liver pathway, the role of the FGFR4/β Klotho signaling, previously described as a target of FXRα could be hypothesized as a regulator of the testicular FXRα pathways as FGF signaling is known to regulate testicular physiology. However, in testis this signaling relies on paracrine interactions between Leydig and Sertoli cells. Indeed, if Fgfr4 is expressed in Leydig cells and regulates steroidogenesis, the FGF ligands are expressed and secreted by the Sertoli cells. Thus regarding the present study as the effects of GW4064 on testicular steroidogenesis as well as on sensitivity to LH/hCG were reproduced on the Leydig MA-10 cell line, it does not support the idea of the involvement of the paracrine FGF-FGFR4 pathway. In addition the expression of Fgfr4 was not altered by the treatment with GW4064 (data not shown). Moreover, we were not able to detect bKlotho in pubertal testis.

Thus, the present study suggests that BA signaling pathways involved might be different between pubertal and adult male mice. Indeed, we have previously shown that BA-exposure in adult leads to infertility. We demonstrate that these effects are strictly mediated by TGR5 [11]. However, it has to be noticed that the short-term impact of BA-diet on steroidogenesis had not been fully studied in adult mice [11]. Thus we could not exclude that such phenomena could also happened in adult mice. It is interesting to note that for other kinds of exposure such as for endocrine disrupters, the window of exposure is critical in regards of the observed phenotypes. Indeed, in utero/neonatal exposure leads to infertility [10] whereas adult exposure leads to transitory germ cell apoptosis [15].

Surprisingly, the invalidation of Shp was not sufficient to avoid the impact of BA exposure on testis physiology. Indeed, if we previously identified that, in adult mice, very short-term exposure to FXRα agonist represses testosterone synthesis *via* SHP, the present work suggests that in longer exposure (chronic exposure, 5-days); FXRα could represses steroidogenesis in a SHP-independent manner. This must be explained by our results demonstrating for the first time that *Dax-1*, a well-known repressor of steroidogenesis within Leydig cells [12], is a direct transcriptional target gene of FXRα. Our data suggest that Dax-1 is involved in the basal repression of steroidogenesis by FXRα. This suggest a first mechanism how FXRα represses steroidogenesis, through the elevation of *Dax-1* and *Shp* expression and their subsequent repressive effects on transcription. Indeed, these two receptors are known repressors of steroidogenesis *via* the interaction with SF1 and/or LRH-1. Interestingly, Dax-1 was previously demonstrated as a target of SF-1 which might be a feedback loop to regulate steroidogenesis. However, in our study, no effect of FXRα signaling was observed on Sf-1 expression. Interestingly, SF-1 and LRH-1 are known to use same response element in sequence of target genes thus dax-1 could be a LRH-1 target gene. However, FXRα represses the expression of Lrh-1, thus it does not feet with induction of Dax-1. We demonstrate here that FXRα directly induces the expression of *Dax-1*. In addition, the regulation of Dax-1 is of interest as it has been recently demonstrated that DAX-1 acts as a co-repressor of FXRα through the competition with co-activators such as SRC-1 and PGC-1α [16]. Combined, these data suggest the existence of a potential negative feedback for a local control of steroidogenesis. This also opens new interesting field of research as *Dax-1* expression is associated with altered reproductive function.

The potential redundancy between SHP and DAX-1 is supported by the fact that even if SHP participates to the response of testicular physiology in case of low LH/CG levels [17], *Shp*^−/−^ males present normal level of LH and normal answer to acute LH surge [21].

The data reported here suggest that the FXRα signaling pathway might also be a regulator of the LH/hCG stimulated testicular steroidogenesis. Indeed, the main striking point of the present work is the identification of the molecular mechanisms of the interaction between FXRα signaling pathway and the hypothalamo-pituitary axis. Our data supported the evidence that the gene encoding the LH-receptor is repressed by FXRα pathways. This demonstrates that BA signaling pathway, at the testicular level, interact with the hypothalomo-pituitary axis in the regulation of testosterone production. This is of importance as, the control of the Leydig cell functions, including steroidogenesis, is predominantly mediated by the hypothalamo-pituitary-gonadal axis via LH/CG [7], which is of major importance for the initiation of sexual maturation.

The interaction of the two defined mechanisms is reinforced by the fact that some impact of LH signaling have been described to be dependent of LRH-1/SF-1 binding sites on the regulatory sequences of steroidogenic genes such as Star [18, 19]. Moreover, there might be a cross-talk between the regulation of *Shp* and *Dax-1* with the altered LH signaling in response to BA as these two targets of FXRα have been shown to be negatively regulated by the LH pathways *via* PKA-AMPK pathways [17, 20].

A role of FXRα in Leydig cells, a question of age? At 12-day-old, FXRα mainly impacts the sensitivity of Leydig cells to LH/hCG, at 20-day-old its signaling alters both basal and LH/hCG stimulated steroidogenesis, and finally at 44dpt the FXRα signaling pathway seems to impact only basal steroidogenesis. Such differences regarding the effects of FXRα on Leydig cell functions could be the results of the regulation of particular target genes. Regarding basal steroidogenesis, either Dax-1 or Shp could be involved. Interestingly, it was previously demonstrated that SHP is expressed in the Leydig cells only since 20-day-old [1]. This could explain in part that FXRα have almost no impact on basal steroidogenesis before 20-day-old.

Interestingly, if FXRα affects LH/hCG sensitivity at 12 days-old, it has only slight impact of basal testosterone levels which was consistent with the lack of effects on germ cell apoptosis rate. These data suggest that at this age the unidentified repressor of Lhcgr in response to activation of FXRα might be induced whereas Dax-1 or Shp might not be induced. Regarding SHP this could be consistent with the fact that at this age SHP is not or low expressed in interstitial space at this age [1].

For instance, we have no clue to explain the impact of FXRα on *Lhcgr* expression as we did not identified the involved repressor. However, our results suggest that the actor(s) invoved in the downregulation of *Lhcgr* in response to FXRα activation could be with a limited expression during prepubertal age.

The present work opens new field of research to better understand physiological and pathological conditions. The remaining question, which will require additional studies, will be to clearly decipher the physiological role of FXRα and BA within Leydig cells. We need to determine in which conditions the FXRα signaling pathway participate to the repression of Leydig steroidogenesis. Several hypotheses could be made. The first point will be to define the endogenous ligand of FXRα within the testis. We have demonstrated that BA are present in the testis in normal conditions [11]. In addition, others defined that steroids derived from androgen catabolism, like androsterone, are potential FXRα ligands [21]. In that line we can make the hypothesis of a testicular feedback loop to repress androgen production as a negative feedback [22]. Combined these data suggest that FXRα could be activated by numerous stimuli within the testis.

Our data reinforce the links between FXRα signaling pathways and steroid metabolism [23, 24]. This is of major importance as endocrine homeostasis is a critical physiological process, as alteration could lead to various diseases. This point has been enlightened in the last decades with the large impact of endocrine disrupters on animal and human health [10, 25]. We thus define FXRα as an important actor of the regulation of testicular physiology during puberty. This is of interest in order to identify the etiology of primary hypogonadism observed in case of liver disorders during puberty period as demonstrated in experimental models [2, 26]. Our results are sustained by the fact that impaired growth and delayed puberty are found in case of progressive familial intrahepatic cholestasis [27]. Combined, the present data and other work [11]support the physiological roles of BA signaling pathways on testicular physiology at different timing during male life.

## MATERIALS AND METHODS

### Ethics statement

This study was conducted in accordance with the current regulations and standards approved by the Animal Care Committee (CEMEA Auvergne) (CE-60-12).

### Animals

C57Bl/6J were purchased from Charles River Laboratories (L'Arbresle, France); *Shp^−/−^* Fxrα−/− and *Tgr5^−/−^* mice have been previously described [11, 10, 28, 29]. The mice used in this study were maintained on a C57BL/6J background and housed in temperature-controlled rooms with 12 hours light/dark cycles. Mice had *ad libitum* access to food and water. 21-days old mice were fed to D04 diet (control) or D04 diet supplemented with 0.5% cholic acid (CA-diet) (SAFE, Augy, France) for 5, 14, 26 or 44 days. As young mice are quite sensitive to CA-diet, they were fed 5 days with CA-diet and 2 following days with the control diet. This sequence was repeated until sacrifice. The choice of this age was to be more efficient in the treatment with CA-diet since from 19 day-old, pups are separated from the mother and thus get fed only by diet. Moreover, it allows to target particular window of the increase of testicular to reach adult levels.

The fertility tests were performed at 44 days after the beginning of the treatment.

For GW4064 experiments, 21-days old mice were daily injected (intra-peritoneal) with 37μl of vehicle (DMSO) or GW4064 (28mg/kg) during 5 days. For specific experiments, mice were injected with hCG (5 IU, equivalent to 1.42mM; Sigma-Aldrich) diluted in NaCl 0.09%.

### Sperm count

The epididymis was harvested. Then the head or tail was mashed and we count head of spermatozoa in order to reflect the sperm production.

### Histology

Testes were collected, paraformaldehyde (PFA)-fixed and embedded in paraffin, and 5 μm-thick sections were prepared and stained with hematoxylin/eosin.

### TUNEL analysis

TUNEL experiments were performed as previously described on 5 μm of testis fixed in PFA 4% [5]. In each testis, at least 100 random seminiferous tubules were counted. Results are expressed as the percentage of tubules with either spermatocytes or spermatids TUNEL-positive.

### Endocrine investigations

Steroids were extracted from testes as previously described [5]. Intra-testicular and plasma levels were measured using commercial kits: testosterone and estradiol (Diagnostic Biochem, London, Canada).

### Real-Time RT-PCR

RNA from testis samples were isolated using Nucleospin RNA (Macherey-nagel, Hoerdt, France). cDNA were synthesized from total RNA with the MMLV reverse transcriptase and random hexamer primers (Promega, Charbonnière Les Bains, France). Real-time PCR measurement of individual cDNAs was performed using SYBR green dye (Master mix Plus for SYBR Assay, Eurogentec, Angers, France) to measure duplex DNA formation with the Eppendorf Realplex system. Sequences of primers are reported in Supplementary Table S1. Standard curves were generated with pools of testis cDNA from animals with different genotypes and/or treatments. Results were analyzed using the ΔΔct method.

### Cotransfection assays

CV1 cells were transfected as described [14]. hDAX-1 promoter-luciferase reporter [30] was kindly provided by Dr E. Lalli. h-NR0B-1-luc construct (50 ng) was added in combination with CMX-mFXRα (15 ng), CMX-mRXRα (15 ng), β-galactosidase (10 ng), and pCMX for a total of 150 ng/well. Ligands were added 6-8 hours later in serum free media. Cells were harvested 14-16 hours later and assayed for luciferase and β-galactosidase activity. Luciferase values were normalized for transfection efficiency using β-galactosidase and expressed as RLU of triplicate assays (mean ± SD).

### siRNA transient transfection

MA10 cells were transfected with small interfering RNA (siRNA) using interferin (Ozyme, Saint Quentin Yvelines, France) in six-well plates (400,000 cells per well). The siRNA directed against *Dax-1*, as well as control siRNA (siGfp), was transfected at 5 ng per well. When 48 hours after the transfection had passed, cells were treated with vehicle (DMSO, 1/1,000) or GW4064. Then, cells were harvested 12 hours later, and mRNA extractions were performed.

### Electrophoretic mobility shift assays

EMSAs were performed as previously described [14].

Experiments were done in vitro translated proteins for FXRα and RXR using the appropriate labeled probe (IR1-hDAX1:5′-CCGCGCCCTTGCCCAGACCGAGGCG-3′). Specificity RXR-FXR of binding was tested by competition with × 100, × 50, and × 20 excess of various unlabeled FXREs (IR1-hFGF19 [13]) or LXRE (LXRE-abca1 [14]); After electrophoresis, gel was dried at 80°C for 1 h and autoradiographed with intensifying screen at −80°C overnight.

### Cell culture experiments

MA10 cells were maintained at 37°C in an atmosphere of 5% CO_2_ with Waymouth (Life Technologies) containing 100 U/ml penicillin and 100 μg/ml streptomycin supplemented with 10% horse serum. On d0, MA10 cells were seeded at 400 × 10^3^ cells per well in 6-well plates and allowed to adhere overnight. The following day, cells were washed twice with 1 × PBS, and the medium without serum was applied with the GW4064 (10^−6^M) or vehicle (DMSO). In some experiments, cells were then treated with vehicle (Nacl) or hCG (2.5nM), Forskolin (10 μM) or 8BrAMPc (100 μM) for 4 hours.

### Statistics

Differences between *two groups* for single point data were determined by Student's *t*-test. For other data obtained two-way analysis of variance was performed. When significant effects were obtained, multiple comparisons were made with Tukey's test. All numerical data are represented as mean ± SE. Significant difference was set at *P*< 0.05.

## SUPPLEMENTARY FIGURES AND TABLE



## Abbreviations

AMPK: AMP activated protein kinase
BA: bile acid
BTB: blood-testicular barrier
CA: cholic acid
cDNA: complementary DNA
Cyp3a25: cytochrome P450, family 3, subfamily a, polypeptide 25
Dax-1: dosage-sensitive sex reversal, adrenal hypoplasia critical region, on chromosome X, gene-1
FXR-α: farnesol X receptor
GPBAR1: G-protein-coupled bile acid receptor 1
hCG: human choriogonadotropin
H&E: hematoxylin and eosin
IHC: immunohistochemistry
IP: intra-peritoneal
IT: intratesticular
LH: luteinizing hormone
mRNA: LRH-1: liver receptor homolog-1; messenger RNA
PFA: para-formaldehyde
PKA: Protein Kinase A
SF-1: Steroidogenic factor-1
SHP: Small heterodimer partner
Star: steroidogenic acute regulatory protein
Sult2a1: sulfotransferase family, cytosolic, 2A, TUNEL, terminal deoxynucleotidyl transferase dUTP nick end labeling
