# Supplementary material for: Bile acid-FXRα pathways regulate male sexual maturation in mice
Source: Oncotarget. 2016 Feb 3;7(15):19468–82. doi: 10.18632/oncotarget.7153 (PMC4991395; doi:10.18632/oncotarget.7153)
Supplement: Supplementary file 1 [file oncotarget-07-19468-s001.pdf]

SUPPLEMENTARY FIGURES AND TABLE

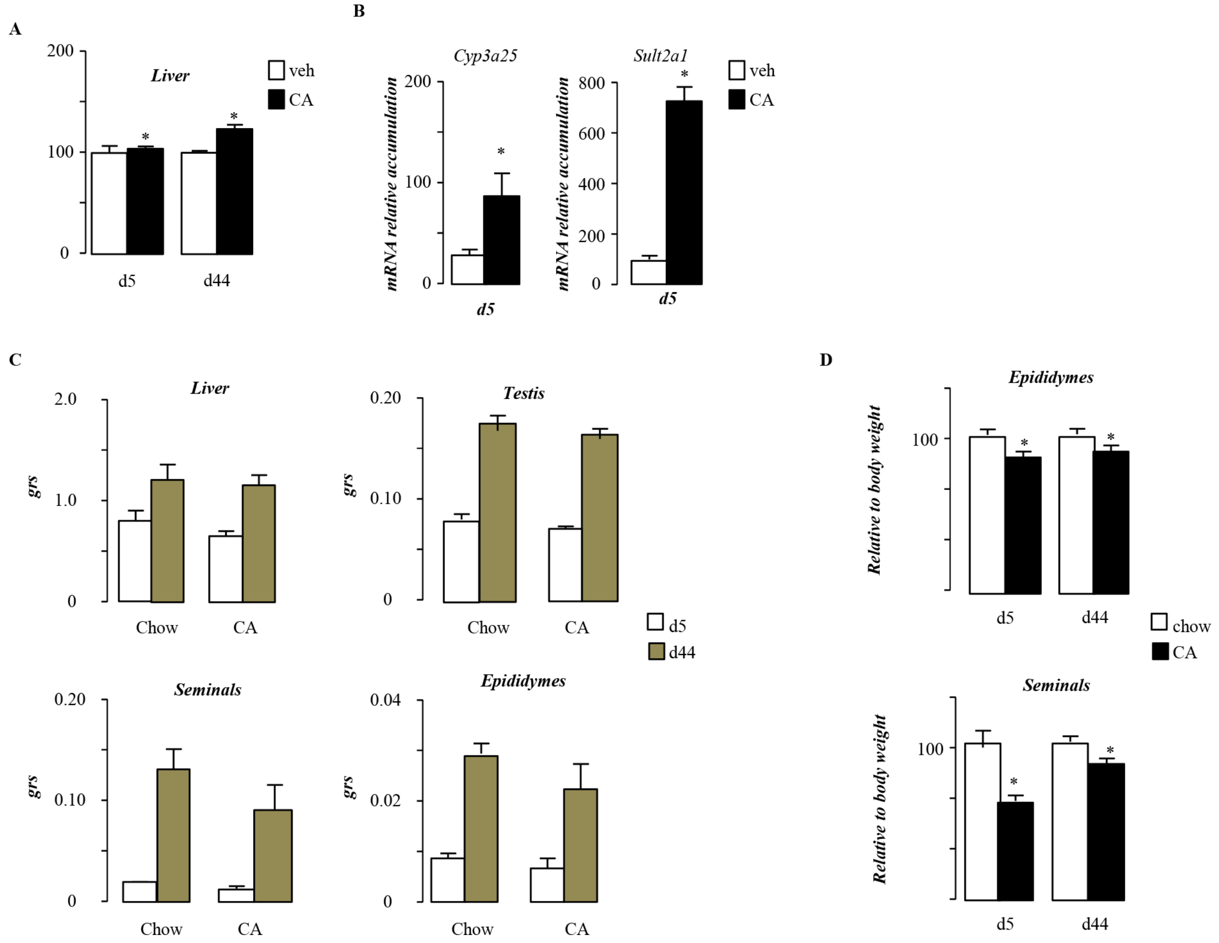

Supplementary Figure S1: A. Liver weights in C57Bl/6J mice fed 5 or 44 days of exposure. **B.** Liver mRNA expression of *Cyp3a25* and *Sult2a1* normalized to *β-actin* levels in whole testis of C57Bl/6J mice fed control or CA diets for 44 days (n= 5 to 6per group). **C.** Liver, testis, seminals and epididymes gross weights at 5 days and 44 days after the beginning of the treatment. **D.** Seminals and epididymes weights relative to body weight at 5 days and 44 days after the beginning of the treatment. In all panels control diet treated mice were arbitrarily fixed at 100% and data are expressed as means ± SEM. Statistical analysis:*, p<0.05; **, p<0.01, ***, p<0.005 *vs*. control diet group.

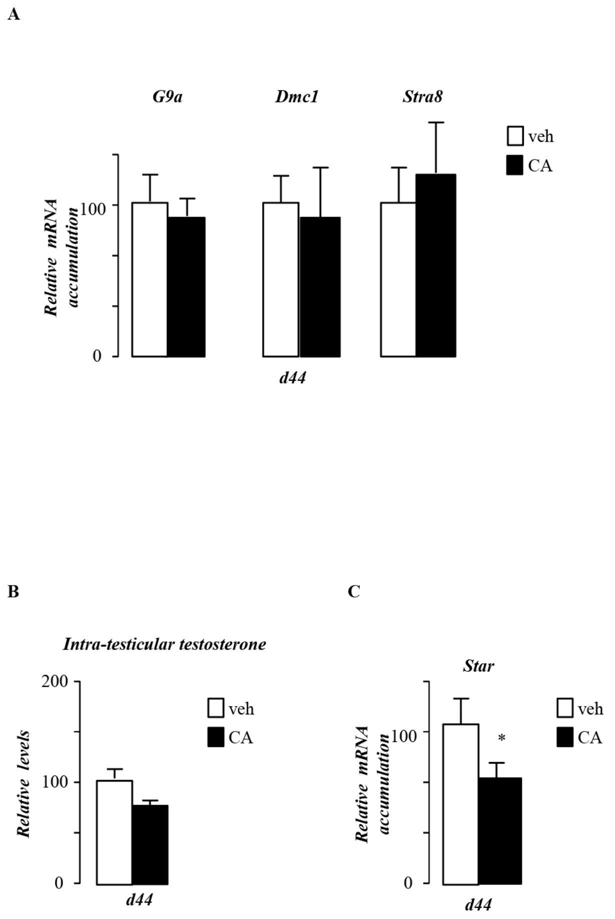

Supplementary Figure S2: A. Testicular mRNA expression of *G9a, Dmc1 and Stra8* normalized to *β-actin* in C57Bl/6J mice fed 44 days with control or CA diets (n= 5–10 per group). **B.** Relative intra-testicular testosterone levels in C57Bl/6J mice fed control or CA diet for 44 days (n= 6–20 per group). **C.** Testicular mRNA expression of *Star* normalized to *β-actin* levels in whole testis of C57Bl/6J mice treated 44 days with CA-diet (n= 5 to 6per group).

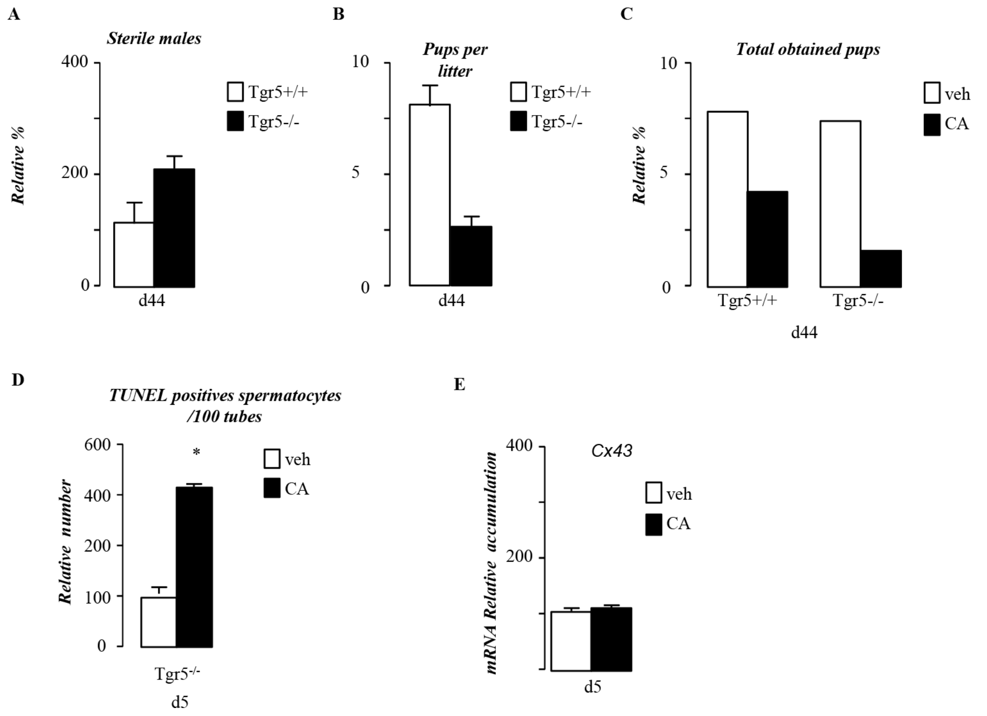

Supplementary Figure S3: **A.** Percentage of infertile males in Tgr5+/+ and Tgr5–/– exposed to CA diet for 44 days. **B.** Number of pups per litter per males in Tgr5+/+ and Tgr5–/– exposed to CA diet for 44 days. **C.** Total number of pups obtained per group in Tgr5+/+ and Tgr5–/ –exposed to control or CA diet for 44 days. **D.** Quantification of TUNEL analyses in Tgr5-/- males fed control or CA diets for 5 days. The number of TUNEL-positive spermatocytes is indicated as the number of positive cells per 100 seminiferous tubules (n= 6–10). **E.** Testicular mRNA expression of *Cx43* normalized to *β-actin* in C57Bl/6J mice fed 5 days with control or CA diets (n= 5–10 per group). In all of the panels data are expressed as the means ± SEM. Statistical analysis:*, p<0.05; **, p<0.01, ***, p<0.005 *vs*. respective control group.

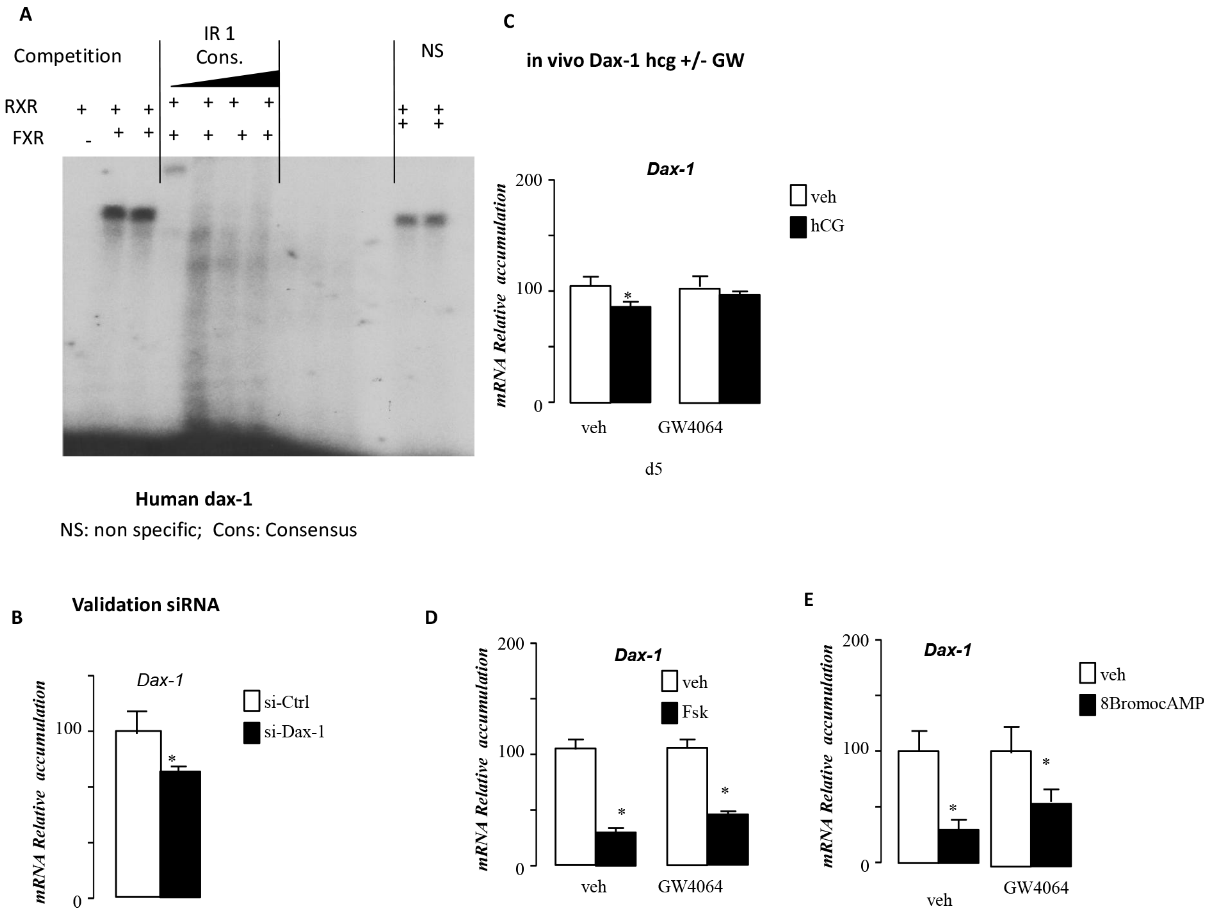

Supplementary Figure S4: A. Electromobility shift assays were performed with 32P]-labeled FXRE and *in vitro*–transcribed hFXRα and/or human RXRα. Competitions were performed using unlabeled nucleotides at 200-fold molar excess of IR1-hFGF19 or a none-specific sequence (LXRE abca1). **B.** mRNA expression of *Dax-1* normalized to *β-actin* levels in MA10 cells after 48 hours of transfection with siRNA-control or siRNA directed against *Dax-1* (n= 10 to 15 per group). **C.** testicular mRNA expression of *Dax-1* in C57Bl/6J mice treated 5 days with vehicle, GW4064 and then 12 hours with veh or hCG; vehicle group was set arbitrarily as 100% (n= 5–15 per group). **D.** mRNA expression of *dax-1* normalized to *β-actin* **levels** in MA10 cells pre-treated 12 h with vehicle or GW4064 and then 4 hours with veh or Fsk (n= 5–15 per group); vehicle group was set arbitrarily as 100%. **E.** mRNA expression of *Dax-1* normalized to *β-actin* levels in MA10 cells pre-treated 12 h with vehicle or GW4064 and then 4 hours with veh or 8bromo-cAMP (n= 5–15 per group); vehicle group was set arbitrarily as 100%.

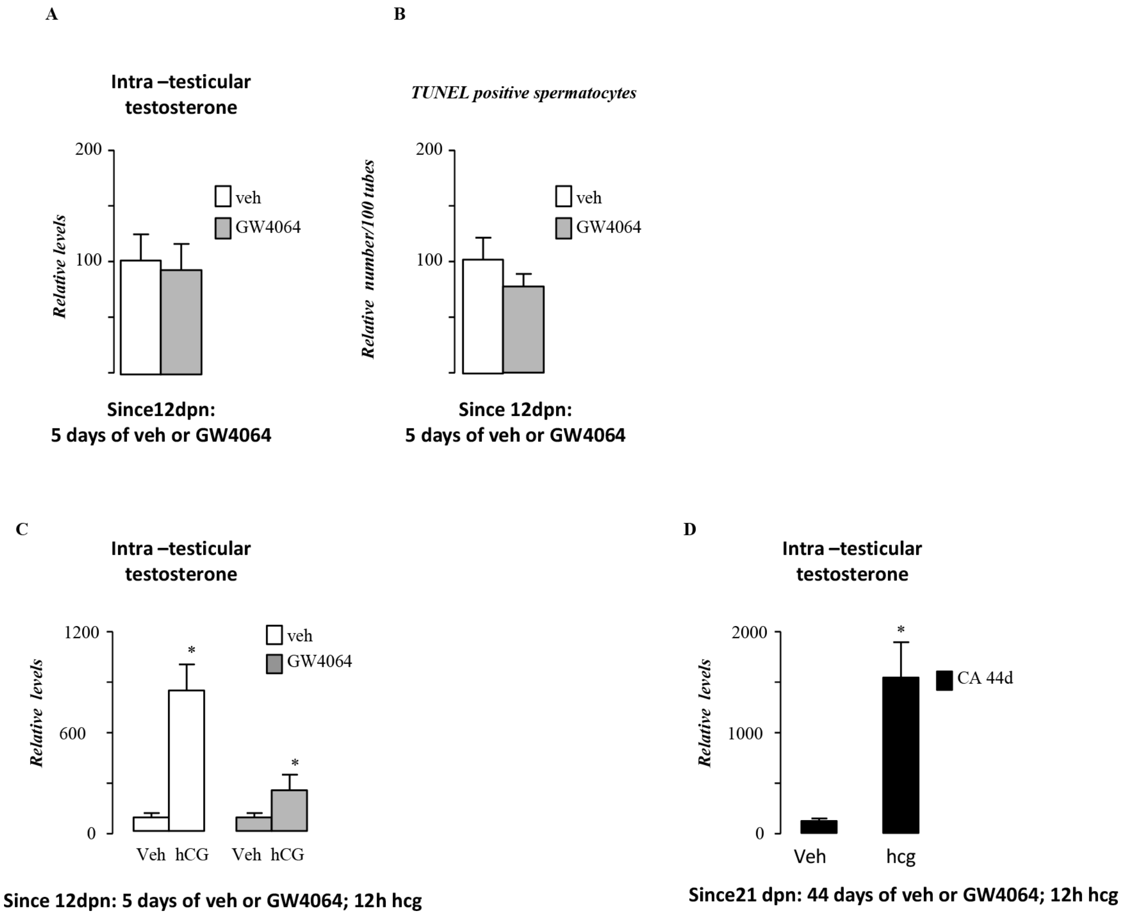

Supplementary Figure S5: A. Relative intra-testicular testosterone levels in 12 days old C57Bl/6J mice fed control GW4064 for 5 days (n= 6–10 per group). **B.** Quantification of TUNEL analyses in 12 days old C57Bl6 males fed control orGW4064 for 5 days. The number of TUNEL-positive spermatocytes is indicated as the number of positive cells per 100 seminiferous tubules (n= 6–10). **C.** Relative intra-testicular testosterone levels in 12 days old C57Bl/6J mice treated 5 days with vehicle, GW4064 and then 12 hours with vehicle or hCG (n= 5–15 per group). **D.** Relative intra-testicular testosterone levels in 21 days old C57Bl/6J mice fed 44 days with control or CA diets and then 12 hours with vehicle or hCG (n= 5–15 per group). In all of the panels Control diet or vehicle treated mice were arbitrarily fixed at 100% and data are expressed as the means ± SEM. Statistical analysis:*, $p<0.05$; **, $p<0.01$, ***, $p<0.005$ *vs*. control diet group.

Supplementary Table S1: Q-PCR Primers list

G9a, Dmc1, Stra8, were from volle et al. [1] ot {11}

Actine	FW	TCATCACTATTGGCAACGAGC
	Rev	AGTTTCATGGATGCCACAGG
Lhcgr	FW	AGCTAATGCCTTTGACAACC
	Rev	GATGGACTCATTATTCATCC
Star	FW	TGTCAAGGAGATCAAGGTCCTG
	Rev	CGATAGGACCTGGTTGATGAT
Nr0b2	FW	CTTTCTGGAGCCTTGAGCTGG
	Rev	GTTGAAGAGGATCGTGCCCTT
Nr5a1	FW	TGCAGAATGGCCGACCAG
	Rev	TGGCGGTAGATGTGGTC
Nr5a2	FW	CTCTTGATTCTCGATCACATTTACC
	Rev	CCAGGAACTTGAGACATACAAACTC
Nr0b1	FW	*TGCACTTCGAGATGATGGAG*
	Rev	*ATCTGCTGGGTTCTCCACTG*
Prm2	FW	CCAGTGAGGGTCAGCACCAG
	Rev	TCTGCAGCCTCTGCGATGCC
